# Identification of miRNAs in cervical mucus as a novel diagnostic marker for cervical neoplasia

**DOI:** 10.1038/s41598-018-25310-1

**Published:** 2018-05-04

**Authors:** Satoshi Kawai, Takuma Fujii, Iwao Kukimoto, Hiroya Yamada, Naoki Yamamoto, Makoto Kuroda, Sayaka Otani, Ryoko Ichikawa, Eiji Nishio, Yutaka Torii, Aya Iwata

**Affiliations:** 1 Department of Obstetrics and Gynecology, Fujita Health University, School of Medicine, 1-98, Dengakugakubo, Toyoake, Aichi 470-1192 Japan; 2 Pathogen Genomics Center, National Institute of Infectious Diseases, Musashi-murayama, 4-7-1, Gakuen, Tokyo, 208-0011 Japan; 3 Department of Hygiene, Fujita Health University, School of Medicine, 1-98, Dengakugakubo, Toyoake, Aichi 470-1192 Japan; 4 Laboratory of Molecular Biology and Histochemistry Joint Research Support Promotion Facility Center for Research Promotion and Support, Fujita Health University, School of Medicine, 1-98, Dengakugakubo, Toyoake, Aichi 470-1192 Japan; 5 Department of Pathology, Fujita Health University, School of Medicine, 1-98, Dengakugakubo, Toyoake, Aichi 470-1192 Japan

## Abstract

microRNAs (miRNAs) play important roles in regulation of gene expression during cervical carcinogenesis. We investigated expression profiles of miRNAs in cervical cancer and its precursor lesions by utilizing cervical mucus. Cervical mucus was collected from 230 patients with a normal cervix, cervical intraepithelial neoplasia (CIN), squamous cell carcinoma (SCC), or adenocarcinoma (AD). The levels of miRNA in the mucus were quantified by miRNA array and real-time reverse-transcriptase polymerase chain reaction (RT-PCR). The performance for detecting diseases was statistically analysed. The expression of miRNAs was further validated in the surgical tissues of enrolled patients. Four miRNAs (miR-126-3p, -20b-5p, -451a, and -144-3p) were significantly up-regulated in SCC and AD compared with normal, and their expression levels correlated with disease severity and high-risk human papillomavirus infection. Receiver operating characteristic curve analyses revealed that the area under the curve values for miR-126-3p, -20b-5p, -451a, and -144-3p were 0.89, 0.90, 0.94, and 0.93, respectively, for SCC plus AD compared with normal, showing high accuracy of cancer detection. Real-time RT-PCR analyses confirmed the expression of these four miRNAs in frozen tissues from cervical cancer. miR-126-3p, -20b-5p, -451a, and -144-3p in cervical mucus are promising biomarkers for cervical cancer and high-grade CINs.

## Introduction

Cervical cancer is the fourth most common cancer in women, with an estimated 528,000 new cases and 265,000 deaths worldwide in 2012^1–3^, and approximately 90% of cervical cancer deaths occur in developing countries. Persistent infection with a high-risk human papillomavirus (HPV), is a necessary cause of cervical cancer development. Despite the fact that organized cytology screening has greatly contributed to decreasing the cervical cancer incidence in developed countries, cytology is not necessarily an ideal tool for screening due to its low sensitivity for detection of high-grade cervical intraepithelial neoplasia (CIN) lesions^4^ and adenocarcinoma^5^. Recently, HPV DNA tests have been introduced into the screening system because these tests exhibit high sensitivity compared to cytology. However, their specificity is inferior because most HPV infections are transient and do not clinically manifest as cervical lesions. Thus, a more specific biomarker needs to be developed^4,6^.

microRNAs (miRNAs), non-coding RNAs of 19–25 nucleotides in length, modulate gene expression by partially pairing with the 3′ untranslated region of their target messenger RNAs^7^, and about two-thirds of human messenger RNAs are thought to be regulated by miRNAs^8^. Approximately 2,500 human miRNAs are currently recorded in the database; miRBase (www.mirbase.org), and specific miRNAs have been described as acting as oncogenes or tumour suppressors^7^. A significant relationship between aberrant expression of miRNAs and HPV infection has also been reported^9,10^.

Aberrant expression of miRNAs in cervical cancer and its precursor lesions were previously investigated^11–13^. In cervical neoplasia tissues, various miRNAs have been found to be differentially expressed compared with matched normal tissues. However, the results of these studies were not always consistent, and were inconclusive as to the roles of miRNAs in supporting or suppressing cervical carcinogenesis. This may be due to differences in the source of materials or methods used for analysis.

To develop miRNAs as a biomarker for cervical cancer and its precursor lesions, it is necessary to select appropriate specimens not only from patients with cervical neoplasia but also from healthy women. An unduly invasive method for specimen collection should be avoided. The miRNAs are overexpressed not only in tissues, but are also secreted into body fluids such as serum, urine, semen, saliva, vaginal fluid^14,15^, and vitreous humour^16^. In patients with bladder cancer, profiling for miRNAs in urine can yield a signature that shows a promising performance^17^. Since bladder tumours are in direct contact with urine, it represents the ideal body fluid for profiling bladder tumours. We assumed that cervical mucus was an ideal material for profiling cervical neoplasia for the same reason. The aim of this study was to examine whether the detection of specific miRNAs could be a biomarker for cervical cancer and its precursor lesions by utilizing cervical mucus in comprehensive microarray analysis. Furthermore, we examined the expression of miRNAs in surgical specimens obtained from enrolled patients to validate the expression of miRNAs in cancer tissues.

## Results

### Identification of miRNAs up-regulated in cervical cancer specimens

**Figure 1:**
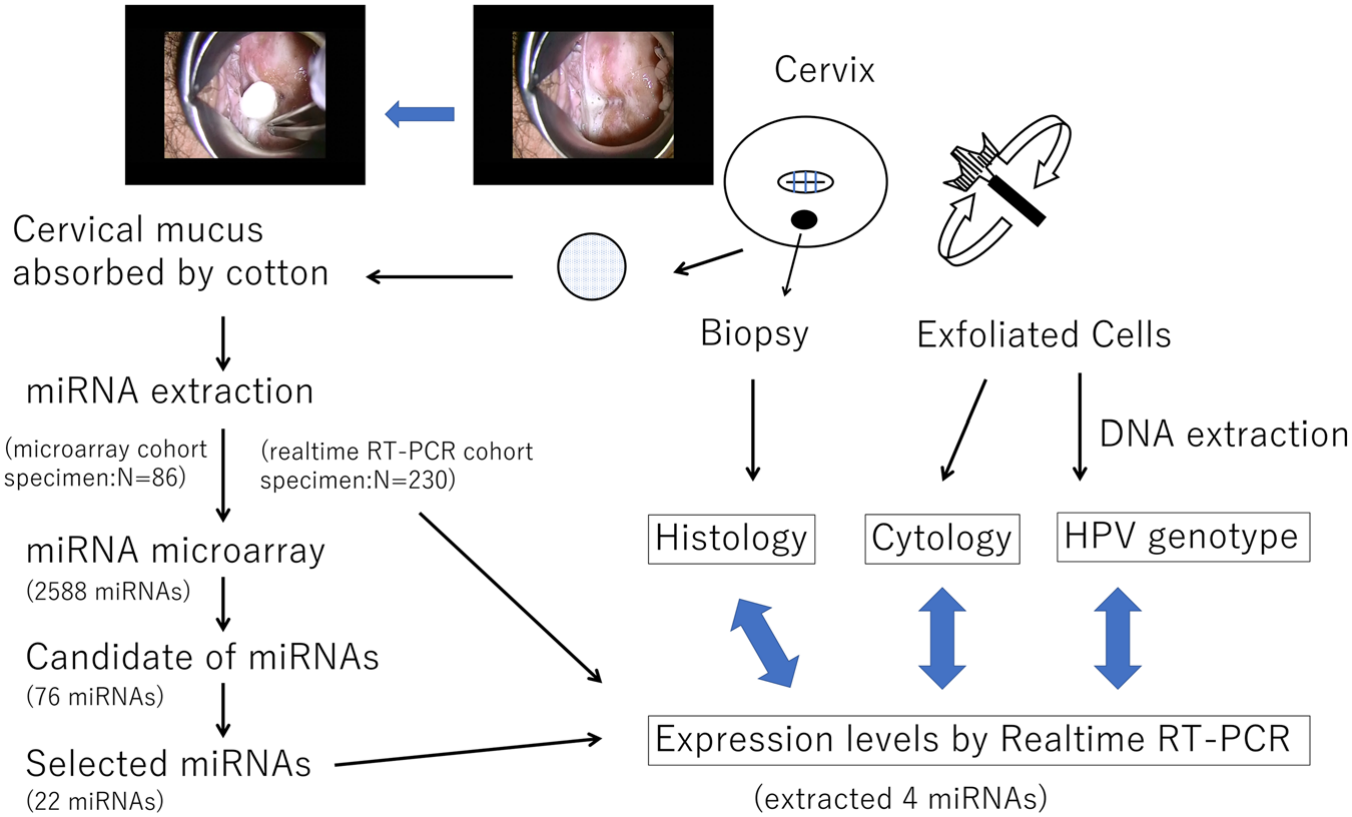
Analytical strategy for identification of miRNAs. A cotton swab was used to collect the cervical mucus. The primary screening was performed on the microarray cohort (n = 86) using a miRNA array. A validation assay with real-time RT-PCR was then performed on the real-time RT-PCR cohort (n = 230). Exfoliated cells and biopsy samples were taken from the cervix. The expression levels of miRNAs by real-time RT-PCR were examined along with information from histology, cytology and HPV genotype.

To identify miRNAs that are up-regulated in cervical cancer compared to normal cervix, we performed miRNA microarray analysis using total RNA extracted from cervical mucus. Among 2588 miRNAs tested, 76 miRNAs were selected as potential biomarker candidates according to the following criteria: the absolute value of the signal intensity was more than 20, and the relative ratio of the squamous cell carcinoma (SCC) vs. normal or adenocarcinoma (AD) vs. normal was more than four. Of the 76 candidates, 22 miRNAs showing relatively high values of signal were further validated by real-time reverse-transcription polymerase chain reaction (RT-PCR) (Fig. 1, Table 1). Among those, four miRNAs (miR-126-3p, -20b-5p, -451a, and -144-3p) exhibited high fold changes of expression. The fold change of SCC vs. normal was 23.4, 8.3, 369.3, and 219.4 for miR-126-3p, -20b-5p, -451a, and -144-3p, respectively. The fold change of AD vs. normal was 9.7, 4.3, 65.6, and 74.1 for miR-126-3p, -20b-5p, -451a, and -144-3p, respectively.

**Table 1:** Association between microarray results and Real-time RT-PCR results for 22 candidates of up-regulated miRNAs.

miRNAs	Microarray					Microarray					Realtime RT-PCR				
group category	global normalization (absolute value)					Fold-change(disease/normal)					Fold-change(disease/normal)				
	Normal	CIN1	CIN3	SCC	AD	Normal	CIN1	CIN3	SCC	AD	Normal	CIN1	CIN3	SCC	AD
hsa-miR-144-3p	14.0	26.5	48.6	577.5	2249.5	1.0	1.9	3.5	41.3	160.9	1.0	0.5	3.2	219.4	74.1
hsa-miR-451a	567.7	1170.8	1931.7	29968.6	52690.1	1.0	2.1	3.4	52.8	92.8	1.0	0.9	5.1	369.3	65.6
hsa-miR-141-3p	44.0	123.2	140.0	275.0	515.5	1.0	2.8	3.2	6.2	11.7	1.0	1.1	1.1	2.3	1.4
hsa-miR-10a-5p	5.0	14.7	32.3	33.4	219.0	1.0	2.9	6.4	6.6	43.5	1.0	1.1	1.1	1.3	2.2
hsa-miR-126-3p	7.5	7.7	11.6	118.4	302.9	1.0	1.0	1.5	15.7	40.1	1.0	1.2	1.2	23.4	9.7
hsa-miR-20b-5p	129.0	93.9	91.9	512.9	1168.0	1.0	0.7	0.7	4.0	9.1	1.0	0.8	0.7	8.3	4.3
hsa-miR-205-5p	318.6	813.3	947.8	2818.2	1189.2	1.0	2.6	3.0	8.8	3.7	1.0	1.6	1.3	2.5	1.3
hsa-let-7f-5p	196.3	207.8	215.3	832.3	1853.0	1.0	1.1	1.1	4.2	9.4	1.0	0.6	0.5	1.7	1.0
hsa-miR-106a-5p	230.2	157.6	154.7	1050.4	2105.3	1.0	0.7	0.7	4.6	9.1	1.0	0.6	0.5	2.3	1.8
hsa-miR-17-5p	218.7	142.5	156.2	963.7	1960.3	1.0	0.7	0.7	4.4	9.0	1.0	0.6	0.5	2.3	1.7
hsa-let-7a-5p	340.0	438.0	504.8	1246.3	2766.3	1.0	1.3	1.5	3.7	8.1	1.0	0.8	0.6	1.8	1.3
hsa-let-7b-5p	259.2	403.4	432.4	1078.2	1874.1	1.0	1.6	1.7	4.2	7.2	1.0	1.2	0.8	1.8	2.1
hsa-let-7c-5p	252.4	375.5	393.6	1020.7	1805.7	1.0	1.5	1.6	4.0	7.2	1.0	0.8	0.5	0.6	0.8
hsa-miR-16-5p	1431.1	883.2	933.7	5119.7	10212.9	1.0	0.6	0.7	3.6	7.1	1.0	0.7	0.8	2.4	1.8
hsa-let-7d-5p	274.0	340.0	343.7	1029.9	1806.1	1.0	1.2	1.3	3.8	6.6	1.0	0.7	0.6	1.6	1.4
hsa-miR-21-5p	556.3	1209.4	1360.6	4170.5	3360.7	1.0	2.2	2.4	7.5	6.0	1.0	1.1	1.7	3.9	1.8
hsa-miR-19b-3p	296.4	180.5	192.6	877.0	1701.3	1.0	0.6	0.6	3.0	5.7	1.0	0.6	0.6	2.1	1.5
hsa-miR-20a-5p	193.4	130.1	137.4	812.6	1746.0	1.0	1.9	1.8	6.7	18.8	1.0	0.7	0.6	2.3	1.4
hsa-miR-15b-5p	186.5	121.0	124.0	562.6	1073.8	1.0	0.6	0.7	3.0	5.8	1.0	1.2	1.3	3.5	4.4
hsa-miR-224-5p	15.2	35.3	40.4	90.9	53.9	1.0	2.3	2.7	6.0	3.6	1.0	1.1	1.4	3.2	1.5
hsa-miR-155-5p	5.4	11.2	10.4	52.8	28.6	1.0	2.1	1.9	9.7	5.3	1.0	2.3	2.1	8.1	2.1
hsa-miR-182-5p	7.8	14.8	14.1	52.2	146.0	1.0	1.9	1.8	6.7	18.8	1.0	0.8	0.7	2.8	2.6

### Associations between miRNA levels and cervical disease progression

**Figure 2:**
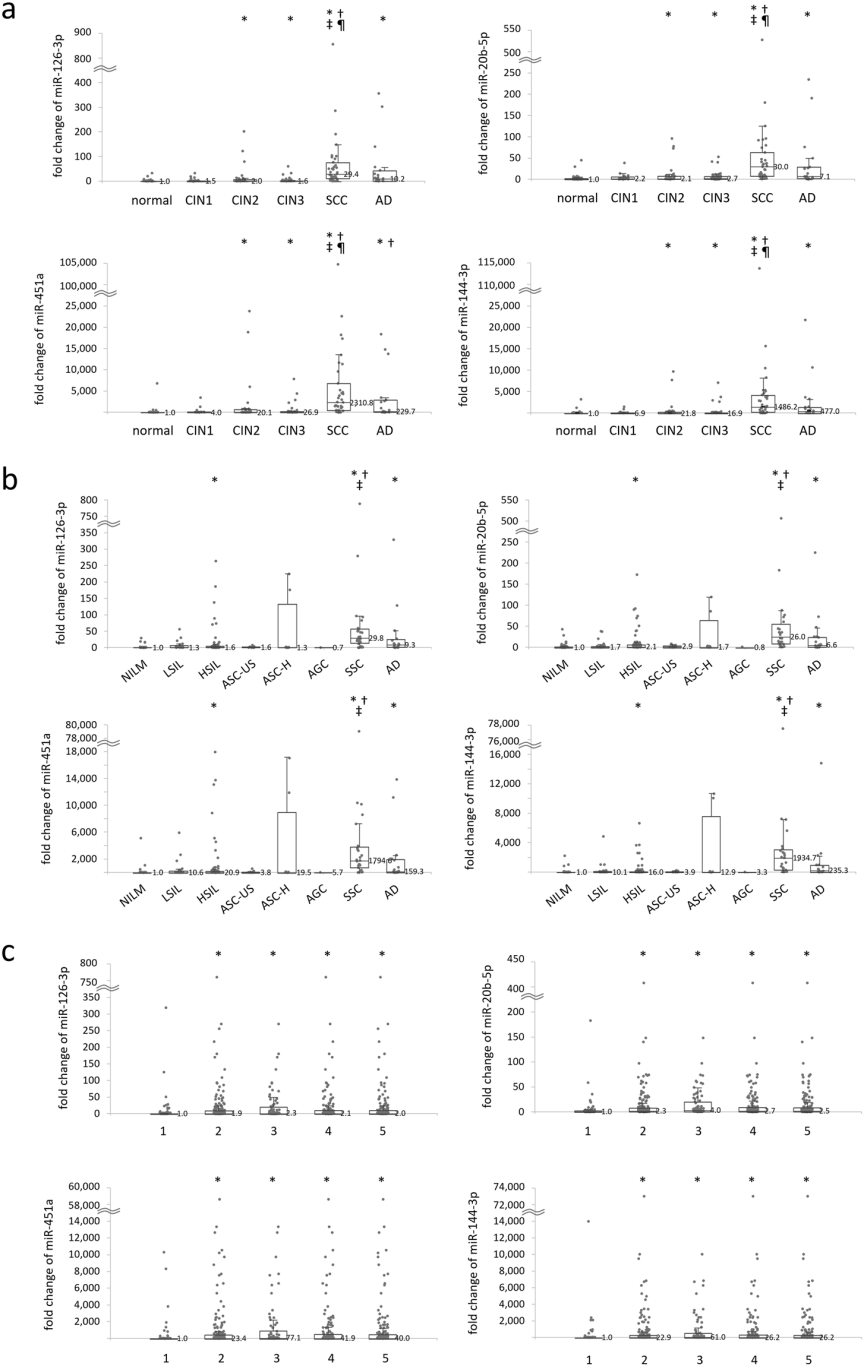
The expression levels of four miRNAs by real-time RT-PCR were correlated with histology (**a**), cytology (**b**) and HPV genotype (**c**) in the real-time RT-PCR cohort. The expression level was corrected against the mean value of the expression level in the normal group (**a**), NILM (**b**) and negative group (**c**). The median value in each group is depicted in the graph. The corrected value of the expression level is indicated on the y-axis and the histology (**a**), cytology (**b**) and the HPV status (**c**) are indicated on the x-axis. (**a**) CIN: cervical intraepithelial neoplasia, SCC: squamous cell carcinoma, AD: adenocarcinoma. The value of these miRNAs significantly increased with the severity of the disease, such as normal, CIN1, 2, 3, and SCC as determined by the Jonckheere–Terpstra trend test. There was a significant difference between normal and CIN2+ as determined by the Kruskal–Wallis test and the Mann–Whitney U test with a Bonferroni correction for all four miRNAs. ^*^P < 0.05 vs normal, ^†^*P* < 0.05 vs CIN1, ^‡^*P* < 0.05 vs CIN2, ^¶^*P* < 0.05 vs CIN3. (**b**) NILM: negative for intraepithelial lesion or malignancy; LSIL: low-grade squamous intraepithelial lesion; HSIL: high-grade squamous intraepithelial lesion; ASC-US: atypical squamous cells of undetermined significance; ASC-H: atypical squamous cells, cannot exclude high-grade squamous intraepithelial lesion; AGC: atypical glandular cells; SCC: squamous cell carcinoma; AD: adenocarcinoma; NILM: negative for intraepithelial lesion or malignancy. The value of these miRNAs significantly increased with the severity of the category through NILM, LSIL, HSIL, and SCC as determined by the Jonckheere–Terpstra trend test. There was a significant difference between NILM and HSIL/SCC as determined by the Kruskal–Wallis test and the Mann–Whitney U test with a Bonferroni correction for all four miRNAs. ^*^P < 0.001 vs NILM, ^†^*P* < 0.001 vs LSIL, ^‡^*P* < 0.001 vs HSIL. (**c**) 1: HPV negative; 2: HPV positive; 3: HPV16/18-positive; 4: seven HPVs (HPV16, 18, 31, 33, 45, 52, and 58); 5: thirteen HPVs (HPV16, 18, 31, 33, 35, 39, 45, 51, 52, 56, 58, 59 and 68). ^*^P < 0.01 vs negative.

We explored associations of the expression levels of the four miRNAs with histology, cervical cytology and HPV infection status. Scatter plots showing the median value of miRNA levels for each specimen with the corresponding average of the *Δ*Ct value for normal tissues (histology), negative for intraepithelial lesions and malignancy (NILM) (cytology), or HPV negative (HPV genotype), are depicted in Fig. 2. In the analysis based on histology, the expression level of these miRNAs significantly increased with the severity of the disease from normal, to CIN1, 2, 3, and SCC as determined by the Jonckheere–Terpstra trend test: p = 1.898 × 10^−13^, 1.255 × 10^−14^, 2.200 × 10^−16^, and 2.200 × 10^−16^, for miR-126-3p, -20b-5p, -451a, and -144-3p, respectively. Of note, in cases of premalignant status such as CIN2 or CIN3, an approximately 16 and 26-fold increase was observed for miR-451a and miR-144-3p, respectively. There was a significant difference across disease categories as determined by the Kruskal–Wallis test: p = 3.268 × 10^−12^, 6.800 × 10^−13^, 5.893 × 10^−16^, and 6.897 × 10^−16^ for miR-126-3p, -20b-5p, -451a, and -144-3p, respectively, and p < 0.005 in each category for all four miRNAs by a Mann–Whitney U test with a Bonferroni correction. There was also a significant difference (p < 0.0001) between normal and AD. On cytology, the value of these miRNAs was significantly increased with the severity of the category such as NILM, low-grade squamous intraepithelial lesion (LSIL), high-grade squamous intraepithelial lesion (HSIL), and SCC as determined by the Jonckheere–Terpstra trend test: p = 1.495 × 10^−9^, 9.318 × 10^−11^, 1.464 × 10^−12^, and 1.473 × 10^−13^ for miR-126-3p, -20b-5p, -451a, and -144-3p, respectively. There was a significant difference between NILM and HSIL/SCC as determined by the Kruskal–Wallis test: p = 4.212 × 10^−9^, 6.385 × 10^−10^, 8.660 × 10^−12^, and 7.800 × 10^−13^ for miR-126-3p, -20b-5p, -451a, and -144-3p, respectively, and p < 0.001 in each category for all four miRNAs by a Mann–Whitney U test with a Bonferroni correction. There was also a significant difference (p < 0.001) between NILM and AD. In contrast, no significant difference in expression levels was observed between NILM and LSIL (p = 0.0929, 0.944, and 0.097 for miR-126-3p, -20b-5p, and -451a, respectively) except for miR-144-3p (p = 0.024), suggesting effective triage for high-grade intraepithelial lesions and cancer.

Regarding HPV genotype, the group positive for HPV16/18 showed the highest levels of these miRNAs: 2.3, 4.0, 27.3 and 61.0 for miR-126-3p, -20b-5p, -451a, and -144-3p, respectively. There was a significant difference between the HPV-negative group and other categories as determined by the Kruskal–Wallis test: p = 0.00039, 9.7 × 10^−6^, 1.52 × 10^−7^, and 5.86 × 10^−8^ for miR-126-3p, -20b-5p, -451a, and -144-3p, respectively. A significant difference (p < 0.001) was also observed between HPV-negative group and other categories by the Mann-Whitney U test with a Bonferroni correction for all four miRNAs.

### Clinical utility of assessing miRNA levels for the detection of cervical lesions

**Figure 3:**
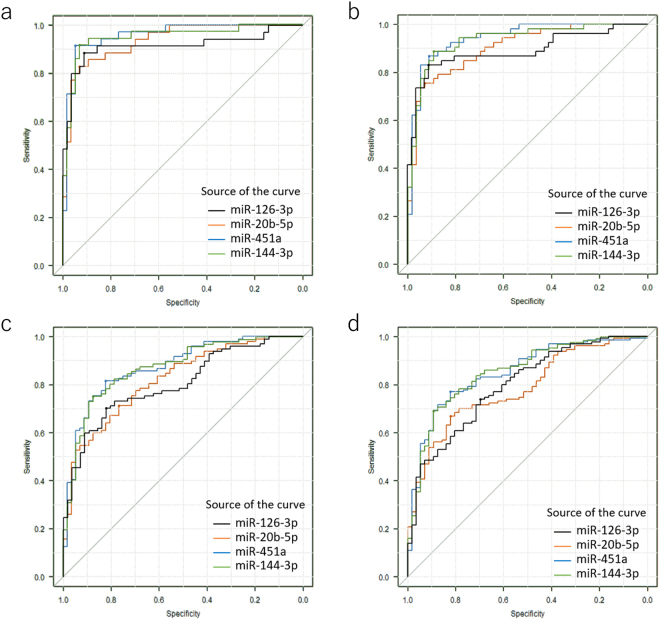
The conventional receiver operating characteristic (ROC) curve to analyse miRNA levels for determining the cut-off points that yielded the highest combined sensitivity and specificity from the Youden index with respect to identifying patients in the normal and disease categories. Sensitivity is indicated on the y-axis and specificity is indicated on the x-axis. Disease category: (**a**) SCC, (**b**) SCC and AD, (**c**) CIN3+, (**d**) CIN2+. The source of the curves is indicated in the graph.

To further evaluate the clinical application of these markers, the cut-off points to detect carcinoma were determined by the Youden index from receiver operating characteristic (ROC) curves (Fig. 3 and Table 2). In SCC, the value for the area under the curve (AUC) was 0.91 (95% confidence interval [CI]: 0.84–0.99), 0.94 (0.89–0.98), 0.96 (0.92–1.00), and 0.95 (0.90–1.00) for miR-126-3p, -20b-5p, -451a, and -144-3p, respectively. The sensitivity was 0.83–0.91 and the specificity was 0.91–0.95. The positive likelihood ratio (PLR) was 9.56–18.20 and the negative likelihood ratio (NLR) was 0.09–0.18. Although the accuracy was inferior to cytology if combined with AD in addition to SCC, the accuracy is acceptable for a screening test^18^. Because performance of the test results showed similarity among the four miRNAs, the concordance rate was examined. There was a strong correlation across the expression levels of the four markers. Agreement was interpreted as near perfect (0.801 < κ < 1.000) among the miRNAs except one combination (0.762; miR-144-3p and -20b-5p in normal vs SCC plus AD) shown in Supplementary Table S2. The highest agreement was observed between miR-451a and -144-3p; 0.930 (normal vs. SCC) and 0.945 (normal vs. ACC plus AD). To further evaluate their performance, we also examined their accuracy to detect the category of the disease including CIN2, CIN3, SCC, and AD (CIN2+) or the category of the disease including CIN3, SCC, and AD (CIN3+) in Table 2 and Fig. 3. The AUC for CIN3+ was 0.80 (95% CI: 0.73–0.87), 0.82 (0.75–0.89), 0.87 (0.81–0.93), and 0.87 (0.81–0.93) for miR-126–3p, -20b-5p, -451a, and -144-3p, respectively. The accuracy of the test was 0.80 or 0.81 to detect CIN3+ by miR-144-3p or miR-451a, respectively. The clinical performance of detecting CIN2+ or CIN3+ was inferior to the detection of cancer; however, the scores showed a moderate accuracy^18^.

**Table 2:** Performance of the miRNA levels to detect cervical neoplasia.

0Normal vs	ROC cut off	AUC	sensitivity	specificity	PLR	NLR	accuracy	PPV	NPV
SCC									
miR-126-3p	3.68	0.91(0.84–0.99)	0.86	0.91	9.56	0.15	0.89	0.86	0.91
miR-20b-5p	5.83	0.94(0.89–0.98)	0.83	0.93	11.86	0.18	0.89	0.88	0.90
miR-451a	116.75	0.96(0.92–1.00)	0.91	0.95	18.20	0.09	0.93	0.91	0.95
miR-144-3p	106.92	0.95(0.90–1.00)	0.89	0.93	12.71	0.12	0.91	0.89	0.93
SCC + AD									
miR-126-3p	3.53	0.89(0.82–0.96)	0.81	0.91	9.00	0.21	0.86	0.90	0.84
miR-20b-5p	5.83	0.90(0.85–0.96)	0.74	0.93	10.57	0.28	0.84	0.91	0.79
miR-451a	37.59	0.94(0.90–0.99)	0.83	0.91	9.22	0.19	0.87	0.90	0.85
miR-144-3p	23.46	0.93(0.89–0.98)	0.87	0.89	7.91	0.15	0.88	0.89	0.88
CIN3+									
miR-126-3p	1.65	0.80(0.73–0.87)	0.69	0.82	3.87	0.38	0.74	0.87	0.61
miR-20b-5p	2.25	0.82(0.75–0.89)	0.71	0.77	3.06	0.38	0.73	0.84	0.61
miR-451a	6.10	0.87(0.81–0.93)	0.80	0.82	4.50	0.24	0.81	0.89	0.71
miR-144-3p	12.92	0.87(0.81–0.93)	0.75	0.88	6.02	0.28	0.80	0.91	0.67
CIN2+									
miR-126-3p	1.65	0.78(0.72–0.85)	0.66	0.82	3.70	0.41	0.71	0.90	0.51
miR-20b-5p	1.55	0.80(0.73–0.86)	0.74	0.70	2.43	0.38	0.73	0.85	0.53
miR-451a	5.95	0.85(0.79–0.91)	0.76	0.82	4.26	0.29	0.78	0.91	0.60
miR-144-3p	17.84	0.85(0.79–0.91)	0.68	0.89	6.39	0.35	0.75	0.94	0.55

Note: ROC: receiver operating characteristic, AUC: area under the curve, PLR: positive likelihood ratio, NLR: negative likelihood ratio. PPV: positive predictive value, NPV: negative predictive value. The cut-off point was determined by the Youden index.

Estimation of AUC: 1.0: perfect match, 1.0–0.9: high accuracy, 0.9–0.7: moderate accuracy, 0.7–0.5: low accuracy, 0.5: chance result^18^.

### Validation of miRNA up-regulation in cervical cancer tissues

**Figure 4:**
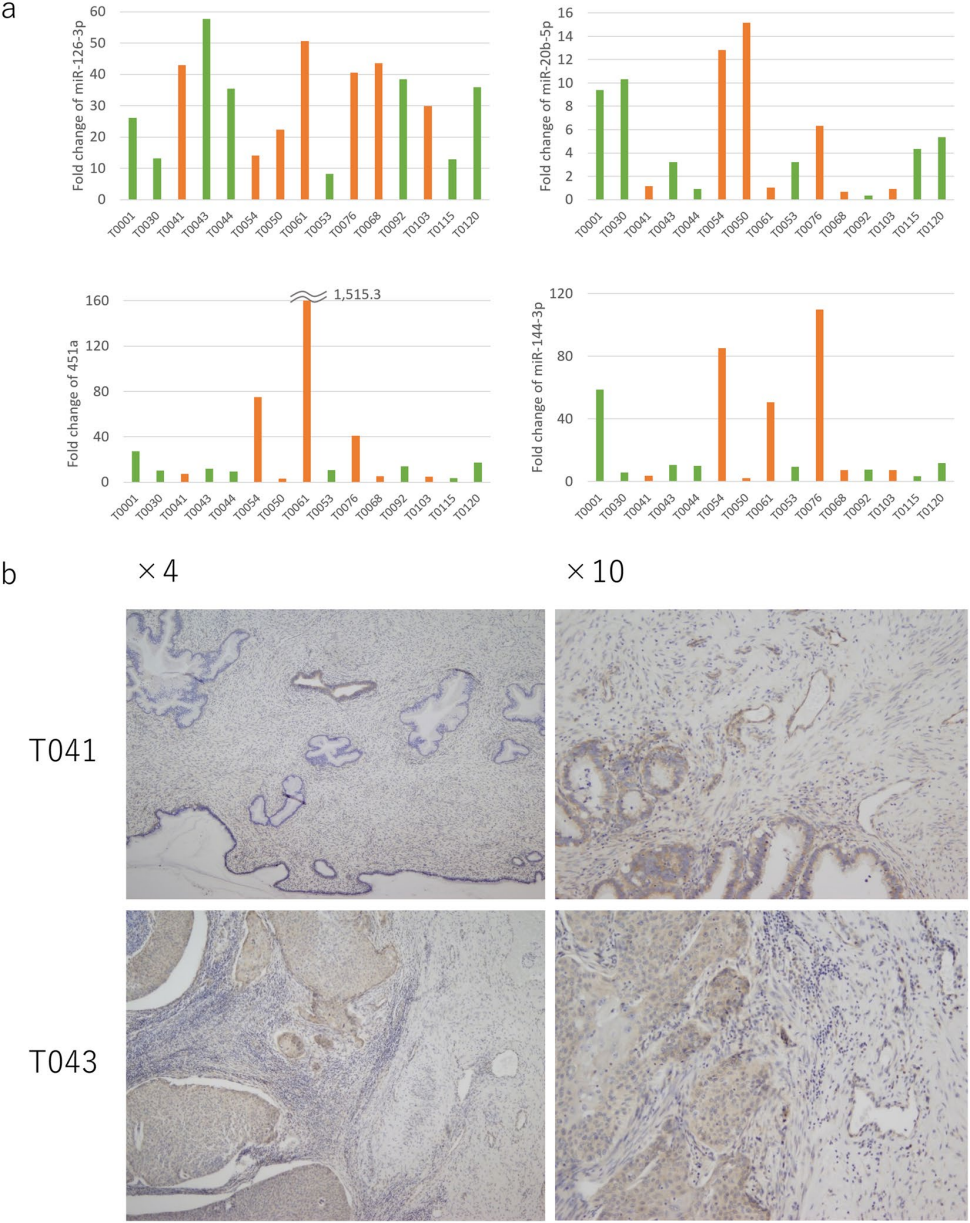
miRNA levels in cervical cancer tissues (**a**). In each surgical specimen, the value of miRNA expression level was corrected for the median value of mucus derived from the normal cervix. The corrected value of the expression level is depicted on the y axis. The available frozen tissues were derived from the enrolled patients in the real-time RT-PCR cohort (tissue ID in Supplementary Table S1). The green and orange bars indicate the tissues from SCC and AD, respectively. SCC: squamous cell carcinoma, AD: adenocarcinoma. Both cervical cancer cells and endothelia in the stroma were stained with a miR-126 probe by *in situ* hybridization. (**b**). *In situ* hybridization with a specific has-mir-126 locked nucleic acid probe was performed on the formalin-fixed paraffin-embedded surgical specimens. miR-126 was highly expressed in the endothelia as well as the cytoplasm of cervical cancer cells. Adenocarcinoma (T041 in Fig. 4a) is shown in the left photo (×4) and the right photo (×10). Normal glands are stained whereas the cytoplasm of cancer cells is stained in T041(×4). Endothelia in the surrounding stroma and cancer cells are stained in T041(×10). Squamous cell carcinoma is shown inT043 (×4) and (×10). The cytoplasm of cancer cells and endothelia in the surrounding stroma are stained.

We next confirmed the up-regulation of miRNAs in cervical tissue samples collected from the enrolled patients. As shown in Fig. 4a, the median ratio of expression levels for cervical cancer vs. normal tissue was 35.4 (range: 8.3–57.7), 3.2 (0.4–15.1), 10.8 (3.1–1515.3), and 9.3 (2.1–109.9) in miR-126-3p, -20b-5p, -451a, and -144-3p, respectively. Of note, a similar expression pattern was observed between miR-144-3p and 451a among the patients with cervical cancer.

### Detection of miR-126-3p in surgical specimens by *in situ* hybridization

Finally, we confirmed the expression of miR-126-3p in surgical specimens of cervical cancer by *in situ* hybridization techniques (Fig. 4b). In 14 of 15 specimens tested, positive staining for miR-126-3p was observed in the endothelia located in the stroma. The cytoplasm of SCC and AD was also positive for miR-126-3p in seven of 14 samples (50%), whereas the adjacent normal squamous cells and glands were all negative.

## Discussion

For analysing miRNAs as a biomarker, it is critical to use a specimen obtained by a less invasive method. Excess cervical mucus or vaginal discharge that interferes with the interpretation of cytology should be removed by a cotton swab and discarded before taking exfoliated cells for cytology. We regarded these discarded materials as an ideal specimen. Cervical mucus contains a mixture of circulating cells and local cellular cells such as secreta from cervical tissues, vaginal discharge, menstrual blood, and cervical exfoliated cells. Therefore, miRNAs in the cervical mucus might be secreted from any of the origins mentioned above. Circulating miRNAs are actively secreted or passively released from dead cells and circulate along with lipoproteins, RNA-binding proteins, and extracellular vesicles. Other sources of miRNAs are plausible, including immune and other blood cells^19^. Although there are a few reports using bio-fluids such as sera^20,21^, this is the first report to identify cervical mucus miRNA profiles in cervical neoplasia. Interestingly, some body fluid specific miRNAs have been reported. Specific aberrant expression of miRNAs has been reported in blood, semen, saliva, vaginal secretions, and menstrual blood^15^. Of note, miR-144-3p is reported to be stably expressed in menstrual blood^14^.

Alternatively, collecting cervical exfoliated cells is also less invasive and they have been widely used in cervical cancer screening, both for HPV and Pap tests^22^. Down-regulated miR-424, miR-218, and miR-375 in exfoliated cells provides a new triage option for HPV-positive women in clinic-based populations. However, down-regulated miRNAs in exfoliated cells are not likely to be useful in screening because of the admixture of normal cells in various proportions. Previous authors have found up-regulated miRNAs including miR-92a and miR-93 but the differences were not statistically significant^22^.

The objective of this study was to determine whether up-regulated miRNAs from profiling of mucus could identify the presence of high-grade CINs and cancer. We comprehensively analysed the levels of mucus miRNAs in patients with cervical neoplasia and a control group by microarray followed by real-time RT-PCR. We selected aberrantly up-regulated four miRNAs from 22 candidates by real-time RT-PCR because the specimens contained an admixture of normal cells (Table 1). On histology or cytology results, four miRNAs, miR-126-3p, -20b-5p, -451a, and -144-3p, were found to be up-regulated in a fashion that was correlated with the severity of CIN and cancer and showed high values for SCC and AD (Fig. 2). In particular, the rate of fold change of miR-451a and 144-3p was extremely high in both AD and SCC. These findings indicate that these miRNAs have promising characteristics as biomarkers because conventional cytology screening is inferior with regard to detecting AD. The levels of these miRNAs were also significantly increased in patients with high-risk HPV infection. It is critical to distinguish between lesions caused by HPV infection (CIN1 or LSIL) and neoplastic lesions (CIN2+ or HSIL) for a triage test. Despite the rate of fold change being significantly associated with high risk HPV infections (Fig. 2), there was no significant difference between normal tissue and CIN1, or between NILM and LSIL, suggesting this assay could be an effective biomarker for identifying high-grade CINs and cancer.

To check the performance of the assay, we examined its accuracy for distinguishing between normal/NILM and cancer (Table 2). For SCC, the AUC range was 0.91–0.96, and the accuracy was 0.89–0.93 with a high PLR (9.56–18.20) and low NLR (0.09–0.18). For detecting CIN3+, the AUC range was 0.80–0.87 and accuracy was 0.73–0.81 with a high PLR (3.06–6.02) and low NLR (0.24–0.38) suggesting these markers are appropriate for a screening test. We have shown that profiling of miRNAs in mucus has a promising performance for detecting cervical neoplasia, including AD, in hospital-based settings.

To check the fidelity of their use as biomarkers, we examined their expression level in frozen cancer tissues. Although there was variety in the expression level individually, we confirmed the miRNAs were expressed in both AD and SCC (Fig. 4). We further examined the localization of the expression of miRNAs in tissues. Unfortunately, we could not detect expression by using miR-20b-5p, 451a, or 144-3p probes purchased from Exiqon. However, we found miR-126-3p was located in the endothelia in the stroma and cytoplasm of cancer cells. We successfully showed a high level of miRNA expression level in the cervical mucus as well as in tissues from the same patients.

In previous reports, inconsistent results of the expression level in cancer have been reported for each miRNA. For example, miR-20b-5p was found to be up-regulated in cervical cancer^23,24^ and HPV-related oropharyngeal cancer^25^, whereas it was down-regulated in papillary thyroid carcinoma^26^. miR-451a suppressed tumour growth in RKO, HeLa, and HUVEC cells^27^. Up-regulation of miR-144-3p inhibited tumour growth in a xenograft tumour model^28^. Down-regulation of miR-144-3p and -126-3p were observed in cervical cancer tissues with lymph node metastasis as compared with cervical cancer without lymph node metastasis^29^. The authors of this study speculated miR-144-3p and-126-3p were suppressors of lymph node metastasis. Interestingly, the expression level of miR-126-3p was down-regulated in cervical cancers^29–32^. In contrast, overexpression of miR-126-3p was observed in the stromal cells, especially the vascular endothelia in cervical cancer tissues^33^. The authors also observed significant overexpression of miR-126-3p in exfoliated cells from patients with cancer compared with the reference. Their results are completely consistent with ours. We assume that this was because they used similar samples due to the fact that mucus contains exfoliated cells. The only difference between our results and theirs is that we observed overexpression of miR-126-3p in both cancer cells and endothelia, possibly due to the differences in the stringent conditions for hybridization (Fig. 4).

We noticed that the diagnostic concordance between miR-451a and miR-144-3p was quite similar on the ROC curve shown in Fig. 3. The concordance rate of normal vs SCC + AD was near perfect, 0.945 between miR-451a and miR-144-3p (Supplementary Table S2). The expression levels of miR-451a and miR-144-3p from frozen tissues of enrolled patients were also similar (Fig. 4a). We found the coding sequence of the genomes of miR-144-3p and miR-451a were surprisingly located on chromosome 17: 28861533–28861618 and chromosome 17: 28861369–28861440, suggesting both miRNAs are located in the same transcript. We then confirmed this transcript was registered as non-coding lincRNA(ENST00000582320.2) in the Ensemble database (http://asia.ensembl.org/Homo_sapiens/Gene/Splice?db=core;g=ENSG00000264066;r=17:28861072-28861966;t=ENST00000582320). miR-451a and miR-144-3p were located in the non-coding lincRNA in which miR-4732-5p and miR-144-5p were also located. Interestingly, miR-4732-5p and miR-144-5p were not eligible for the criteria of up-regulation in the primary microarray analysis (Supplementary Table S3). This suggests that clustering miRNAs are not always up-regulated even in the same transcript.

There are some limitations to this study. Specimens from patients were collected in the university hospital. There may be some selection bias for the enrolled population. In particular, we recognize that the cytology results were not obtained from the general population. Further investigation will be required using a much larger dataset obtained from population-based screening, including many healthy women. In future, the performance of the test in terms of accuracy should be compared with cytology or HPV test results obtained from population-based screening. For establishing a screening test, specific endogenous controls or spike-in exogenous controls are necessary. Transportation or preservation of specimens is critical in a clinical setting to conserve the quality of miRNAs.

Despite its limitations, our study has some strengths. Since there were no profiling miRNAs in the cervical mucus, we analysed the levels of miRNA from patients with normal, precursor lesions, or cervical cancer, taken under identical conditions. We also obtained information such as histology, cytology, and HPV genotype from the same patients as much as possible. Using a normal control as a reference was critical to distinguishing normal from abnormal. The normal histology results in this study also contained NILM on cytology or HPV genotype negative results (Supplementary Table S1). For biological validation, the expression levels of miRNAs in tissues was also examined from the enrolled patients.

In conclusion, this is the first report to identify four specific miRNAs (miR-126-3p, -20b-5p, -451a, and -144-3p) in cervical mucus as promising biomarkers for detecting cervical cancer, especially AD and high-grade CINs.

## Methods

### Study subjects

We performed a series of experiments to compare miRNA profiles with clinical parameters such as histology, cytology, and HPV infection. The estimation of its performance as a biomarker depends on the components of the disease category in the enrolled patients. We based the disease category on the histology, cytology, and HPV genotype identified in each patient. The exclusion criteria were an age of <20 years, pregnancy, previous treatment with chemotherapy, radiation, or surgery for any cancer or CIN. The three tests were independent; however, the outcomes of each test were correlated with one another in clinical situations. Therefore, patients with remarkably inconsistent results between cytology and histology were excluded due to the possibility of sampling error.

The study protocol was approved by the ethical committee of Fujita Health University (HM16-080, HM16-343). Written informed consent was obtained from each patient. All experiments were performed in accordance with the approved guidelines and regulations. The cervical mucus for miRNA was collected by a 1-cm diameter cotton swab and stored at −80 °C. Exfoliated cells were then collected to determine cytology and HPV genotype. In most cases, the specimens for biopsy, Pap smear, and HPV genotype were collected simultaneously but in some cases various samples collected within two months were used in the analysis.

The histopathological diagnosis of CIN was based on the Richart classification. Cytological results were interpreted according to the Bethesda 2001 system. HPV genotype assays were performed by PCR with PGMY primers followed by reverse line blot hybridization. This assay can detect 31 HPV genotypes including HPV 6, 11, 16, 18, 26, 31, 33, 34, 35, 39, 40, 42, 44, 45, 51, 52, 53, 54, 55, 56, 57, 58, 59, 66, 68, 69, 70, 73, 82, 83, and 84^34^.

### Sample groups and strategy

For primary screening, we employed non-amplified RNA samples (microarray cohort specimen) for microarray analysis of gene expression using a 3D-Gene miRNA microarray platform (Toray, Kamakura, Japan) including 2588 miRNAs based on miRBase version release 21 (http://www.mirbase.org/) because of its high sensitivity^35,36^. The candidate miRNAs were then validated by real-time RT-PCR using a real-time PCR cohort specimen. The microarray cohort was comprised of specimens from 86 patients diagnosed with normal or cervical neoplasia. The samples were examined for the expression of miRNA using the 3D-Gene miRNA microarray platform (Toray) (Fig. 1). The samples were classified into normal (n = 16), CIN1 (n = 11), CIN3 (n = 29), SCC (n = 19), and AD (n = 11). The median age in each group was 38, 37, 34, 56, and 47 years, respectively.

Next, in the real-time RT-PCR cohort, the mucus of the 230 patients was examined for the expression of candidate miRNAs (Supplementary Table S1). This cohort included the microarray cohort (n = 86). The histology was classified into normal (n = 56), CIN1 (n = 19), CIN2 (n = 33), CIN3 (n = 43), SCC (n = 35), and AD (n = 19). The median age in each of these groups was 36, 39, 38, 36, 54, and 47 years, respectively. The cytology was classified into NILM (n = 39), atypical squamous cells of undetermined significance (n = 10), LSIL (n = 21), HSIL (n = 75), atypical squamous cells, cannot exclude HSIL (n = 8), atypical glandular cells (n = 1), SCC (n = 24), and AD (n = 16). The median age in each of these groups was 36, 37, 37, 37, 41, 29, 59, and 49 years, respectively. The HPV genotype was classified as positive (n = 164) or negative (n = 66). The median age in these two categories was 39 and 37 years, respectively. Three groups of HPV genotypes were selected for further analysis: (1) HPV16/18, (2) the most common seven high-risk HPV genotypes in Japan (HPV 16, 18, 31, 33, 45, 52, and 58), and (3) 13 genotypes (HPV 16, 18, 31, 33, 35, 39, 45, 51, 52, 56, 58, 59, and 68) found worldwide.

### miRNA microarray analysis

A total of 2 μg of extracted total RNA (see the RNA extraction protocol in Supplementary Information) from the pooled samples in each disease category was prepared and 250 ng of this was labelled with a 3D-Gene miRNA labelling kit (Toray) followed by hybridizing onto 3D-Gene Human miRNA Oligo chips (Toray). The annotation and oligonucleotide sequences of the probes were confirmed by the miRBase miRNA database Release 21. After stringent washing, fluorescent signals were scanned with the 3D-Gene Scanner (Toray) and analysed using 3D-Gene Extraction software (Toray Industries). The raw data from each spot were normalized by substitution with a mean intensity of the background signal determined by all blank spots’ signal intensities and 95% confidence intervals. Measurements of spots with signal intensities greater than 2 standard deviations of the background signal intensity were considered to be valid. The relative expression level of a given miRNA was calculated by comparing the signal intensities of the valid spots throughout the microarray experiments. The normalized data were globally normalized per array, such that the median of the signal intensity was adjusted to 25. The value of each gene was normalized by a method such that the median of disease category/normal ratio was equalized to one. Our data is available in the Gene Expression Omnibus database (GEO; https://www.ncbi.nlm.nih.gov/geo/) with the accession number as GSE105409.

### Real-time RT- PCR

In the second cohort, the candidate miRNAs were validated by real-time RT-PCR. microRNAs were quantified using TaqMan® MicroRNA Assays (Thermo Fisher Scientific, Waltham, MA, USA) with modifications. Briefly, 5.34 ng of total RNA was reverse transcribed (RT) by a TaqMan® MicroRNA RT Kit. The total volume of 8 µL RT reactions contained 10× RT buffer, 0.08 µL of 100 mM dNTPs with dTTP, 0.1 µL of RNase-inhibitor (20 units/µL), 0.53 µL of MultiScribe™ Reverse Transcriptase (50 units/µL), 1.6 µL of each of the microRNA specific stem-loop primers (hsa-miR-20b, 001014; hsa-miR-126, 002228; hsa-miR-451a, 001141; hsa-miR-144, 002676; and RNU48 as a Mature miRNA Control, 001006; Thermo Fisher Scientific) and 2.67 µL (5.34 ng as the template) of input RNA. The mixture was incubated at 16 °C for 30 min, 42 °C for 30 min, and 85 °C for 5 min. Subsequently, quantitative real time-PCR was performed using a 7900 Real-Time PCR system (Thermo Fisher Scientific). For each 20 µL PCR reaction, 20× TaqMan® MicroRNA Assays containing PCR primers and probes (5′-FAM and 3′-TAMRA), 1.5 µL of RT product, and 10 µL of 2× TaqMan® Gene Expression Master Mix (Thermo Fisher Scientific) were mixed together. The reaction was first incubated at 50 °C for 2 min and 95 °C for 10 min, followed by 50 cycles of 95 °C for 15 sec and 60 °C for 1 min. Data were analysed with RQ Manager 1.2 (Thermo Fisher Scientific) with the automatic Ct setting for adapting baselines and thresholds for Ct determination. Relative fold changes were determined from the Ct values with the 2^−*ΔΔ*Ct^ method. Data were normalized to RNU48 to account for possible differences in the amount of starting RNA^37^.

### miRNA *in situ* hybridization

See Supplementary Information.

### Statistical analysis

The Jonckheere–Terpstra trend test was used to determine if there was a statistically significant trend among disease category groups. We used Kruskal–Wallis one-way analysis of variance on ranks to compare overall differences among disease category groups. We compared the fold value of all groups using two-tailed Mann–Whitney U tests with Bonferroni corrections. A multiple pairwise planned comparison was made. We defined P < 0.05 as significant. We used a conventional ROC curve with the Youden index to analyse miRNA levels to determine the cut-off points that yielded the highest combined sensitivity and specificity with respect to distinguishing patients with cancer from those with normal histology. Interpretation of the AUC was as follows: 1.0, perfect match; 1.0–0.9, high accuracy; 0.9–0.7, moderate accuracy; 0.7–0.5, low accuracy; and 0.5, chance result^18^.

On histology, we defined normal as negative and SCC or AD as a positive on screening. Sensitivity, specificity, and positive and negative likelihood were calculated with standard formulas. Agreement of miRNAs with cut-off values between paired miRNAs was evaluated with Cohen’s kappa (k) coefficient. Alternatively, we calculated the accuracy as the performance of the screening test to differentiate CIN2+ or CIN3+ from normal. The statistical analysis was undertaken with EZR (Saitama Medical Center, Jichi Medical University, Saitama, Japan), which is a graphical user interface for R (The R Foundation for Statistical Computing, Vienna, Austria). More precisely, it is a modified version of R commander (version 1.35) designed to perform statistical functions frequently used in biostatistics^38^.

## Electronic supplementary material

supplementary infomation,supplementary table1,2,3
