# Supplementary material for: Identification of miRNAs in cervical mucus as a novel diagnostic marker for cervical neoplasia
Source: Sci Rep. 2018 May 4;8:7070. doi: 10.1038/s41598-018-25310-1 (PMC5935744; doi:10.1038/s41598-018-25310-1)
Supplement: Supplementary file 1 — supplementary infomation,supplementary table1,2,3 [file 41598_2018_25310_MOESM1_ESM.pdf]

Supplementary Information

Identification of miRNAs in cervical mucus as a novel diagnostic marker for cervical neoplasia

Satoshi Kawai, Takuma Fujii, Iwao Kukimoto, Hiroya Yamada, Naoki Yamamoto, Makoto Kuroda, Sayaka Otani, Ryoko Ichikawa, Eiji Nishio, Yutaka Torii, Aya Iwata

Supplementary Methods

RNA extraction from cotton swabs and surgical tissues

Total RNA from cotton swabs was extracted with the miRNeasy Mini Kit (QIAGEN GmbH, Hilden, Germany). Briefly, each cotton swab was soaked in 900 µL of QIAzol Lysis Reagent. The median yield of recovery of total RNA was 11.6 µg (range,0.9–94.1) from each patient.

Freshly excised tumours (n = 15) were collected at the time of surgery (Supplementary Table S1) and immediately stored in 700 µL of RNAlater Stabilization Solution (Thermo Fisher Scientific). After removal of the solution, the tissues were stored at −80°C. TRIzol reagent (Thermo Fisher Scientific) was used to isolate total RNA including miRNAs from 100 mg tissue samples according to the manufacturer’s instructions. The isolated RNA was dissolved in 100–200 µL RNase-free water depending on the volume of precipitation. The RNA concentration and purity were determined by optical density measurement using NanoVue (GE Healthcare UK Ltd., Little Chalfont, England).

miRNA in situ hybridization

The cellular localization of miRNA was examined in the 15 surgical formalin-fi,xed paraffin-embedded specimens corresponding to patient samples used in the realtime PCR analysis. Double (3′and 5′) digoxigenin-labeled

miRCURY LNA detection probes (Exiqon, Vedbaek, Denmark) were used for visualization of the miRNA hsa-miR-126-3p (probe sequence: GCATTATTACTCACGGTACGA, Tm = 84°C) and U6 (CACGAATTTGCGTGTCATCCTT, Tm = 84°C) as a positive control. For in situ hybridization, 3-μm-thick sections of formalin-fixed paraffin-embedded tissues were mounted on Superfrost glass slides and deparaffinized in xylene baths, followed by serial dilutions of ethanol and PBS. The slides were then immersed in 0.3% H_2O_2 for 10 min at room temperature, washed twice with PBS, digested with 20 μg/mL proteinase K (Exiqon) at 37°C for 15 min, and washed twice with PBS.

The slides were pre-hybridized at 53°C for 30 min in hybridization buffer (Exiqon) and then hybridized at 53°C for 1 h with 40 nM probes for miR-126-3p or 20 nM probes for U6 in hybridization buffer. After saline sodium citrate stringent washes, the slides were blocked with Protein Block Serum-free (Agilent Technologies, Santa Clara, CA, US) and incubated with anti-digoxigenin-POD, Fab fragments from sheep (Roche, Mannheim, Germany) diluted to 1:100 at 37°C for 1 h. POD signals were visualized using the Liquid DAB + Substrate Chromogen System (Agilent Technologies), and the slides were stained with hematoxylin for nuclear staining. The slides were then dehydrated and mounted with a coverslip. A microscope (Power BX-51; Olympus, Tokyo, Japan) was used for observation.

Supplementary table S1. Clinical profile of the realtime PCR cohort (n = 230) by disease category

Identification of miRNAs in cervical mucus as a novel diagnostic marker for cervical neoplasia

Satoshi Kawai, Takuma Fujii, Iwao Kukimoto, Hiroya Yamada, Naoki Yamamoto, Makoto Kuroda, Sayaka Otani, Ryoko Ichikawa, Eiji Nishio, Yutaka Torii, Aya Iwata

normal (N=56)

ID	age	cytology	HPV
Sc-0033	29	NILM	negative
Sc-0054	31	NILM	negative
Sc-0065	41	NILM	negative
Sc-0078	37	NILM	negative
Sc-0080	26	NILM	negative
Sc-0081	31	NILM	negative
Sc-0082	27	NILM	negative
Sc-0086	39	NILM	negative
Sc-0098	38	NILM	negative
Sc-0099	43	NILM	negative
Sc-0118	39	NILM	negative
Sc-0141	39	NILM	negative
Sc-0181	36	NILM	negative
Sc-0270	31	NILM	negative
Sc-0285	31	NILM	negative
Sc-0340	39	NILM	negative
Sc-0018	36	NILM	negative
Sc-0058	34	NILM	negative
Sc-0067	44	NILM	negative
Sc-0075	38	NILM	negative
Sc-0090	39	NILM	negative
Sc-0093	34	NILM	negative
Sc-0109	31	NILM	negative
Sc-0125	36	NILM	negative
Sc-0147	35	NILM	negative
Sc-0165	39	NILM	negative
Sc-0182	32	NILM	negative
Sc-0215	34	NILM	negative
Sc-0219	41	NILM	negative
Sc-0226	44	NILM	negative
Sc-0228	43	NILM	negative
Sc-0256	35	NILM	negative
Sc-0271	32	NILM	negative
Sc-0273	37	NILM	negative
Sc-0310	37	NILM	negative
Sc-0313	43	NILM	negative
Sc-0333	35	NILM	negative
Sc-0047	30	ND	negative
Sc-0059	38	ND	negative
Sc-0068	39	ND	negative
Sc-0069	30	ND	negative
Sc-0106	37	ND	negative
Sc-0142	41	ND	negative
Sc-0155	35	ND	negative
Sc-0157	35	ND	negative
Sc-0164	43	ND	negative
Sc-0173	35	ND	negative
Sc-0211	28	ND	negative
Sc-0218	35	ND	negative
Sc-0275	41	ND	negative
Sc-0304	37	ND	negative
Sc-0317	43	ND	negative
Sc-0320	26	ND	negative
Sc-0345	36	ND	negative
Sc-0346	31	ND	negative
Sc-0222	42	ND	negative

CIN1 (N=19)

ID	age	cytology	HPV
Sc-0406	68	ASC-H	53, 58, 66
Sc-0027	39	ASC-US	52
Sc-0032	26	ASC-US	39
Sc-0073	53	ASC-US	68
Sc-0122	66	ASC-US	58
Sc-0341	28	ASC-US	16, 18, 53, 69, 82
Sc-0378	30	ASC-US	16, 68
Sc-0417	51	ASC-US	53, 68
Sc-0174	48	HSIL	16, 31, 52
Sc-0034	37	LSIL	45
Sc-0025	25	LSIL	11
Sc-0048	34	LSIL	55
Sc-0245	35	LSIL	39
Sc-0419	55	LSIL	56
Sc-0114	43	NILM	68
Sc-0146	24	NILM	84
Sc-0175	28	NILM	42
Sc-0007	42	NILM	negative
Sc-0185	43	NILM	56

CIN2 (N=33)

ID	age	cytology	HPV
Sc-0260	32	ASC-H	16
Sc-0375	42	ASC-H	52
Sc-0382	47	ASC-H	33, 55
Sc-0001	45	HSIL	16
Sc-0002	38	HSIL	33
Sc-0014	42	HSIL	33
Sc-0023	31	HSIL	52, 53
Sc-0026	33	HSIL	59
Sc-0097	28	HSIL	58
Sc-0112	29	HSIL	16, 58
Sc-0116	39	HSIL	33, 58
Sc-0121	51	HSIL	16, 39
Sc-0143	33	HSIL	52
Sc-0177	38	HSIL	16
Sc-0210	30	HSIL	18, 66
Sc-0241	42	HSIL	58
Sc-0292	35	HSIL	16, 58
Sc-0325	43	HSIL	16, 35
Sc-0344	31	HSIL	16, 53
Sc-0361	24	HSIL	16
Sc-0376	60	HSIL	52
Sc-0385	40	HSIL	negative
Sc-0389	31	HSIL	52
Sc-0416	33	HSIL	6, 52
Sc-0423	42	HSIL	16
Sc-0438	25	HSIL	51, 58
Sc-0004	28	LSIL	18, 45
Sc-0024	38	LSIL	52
Sc-0041	39	LSIL	16
Sc-0057	49	LSIL	58
Sc-0117	31	LSIL	51
Sc-0003	41	NILM	52
Sc-0187	40	ND	52

CIN3 (N=43)

ID	age	cytology	HPV
Sc-0019	51	AD	52
Sc-0428	40	ASC-H	52
Sc-0062	35	ASC-US	16
Sc-0006	30	HSIL	16
Sc-0010	29	HSIL	16
Sc-0013	27	HSIL	16, 18, 31, 53
Sc-0016	34	HSIL	52
Sc-0021	30	HSIL	16
Sc-0028	34	HSIL	16
Sc-0030	52	HSIL	16
Sc-0035	25	HSIL	16, 45, 51, 52
Sc-0036	37	HSIL	58
Sc-0037	37	HSIL	31, 58
Sc-0039	42	HSIL	33
Sc-0040	34	HSIL	58
Sc-0046	46	HSIL	35
Sc-0091	31	HSIL	16
Sc-0094	30	HSIL	31, 42
Sc-0096	37	HSIL	52
Sc-0135	34	HSIL	58
Sc-0145	41	HSIL	16, 52
Sc-0193	28	HSIL	53, 82
Sc-0244	39	HSIL	6, 52, 69
Sc-0262	37	HSIL	52
Sc-0330	35	HSIL	82
Sc-0055	42	HSIL	52
Sc-0358	33	HSIL	58
Sc-0359	73	HSIL	58
Sc-0366	29	HSIL	16
Sc-0372	40	HSIL	51
Sc-0387	67	HSIL	31
Sc-0392	33	HSIL	16
Sc-0396	43	HSIL	58
Sc-0404	35	HSIL	16, 52
Sc-0412	57	HSIL	52
Sc-0422	36	HSIL	16
Sc-0430	37	HSIL	16, 18
Sc-0433	48	HSIL	68
Sc-0151	39	LSIL	16
Sc-0077	33	LSIL	42
Sc-0113	33	SCC	16
Sc-0420	54	SCC	52
Sc-0356	21	ND	16, 39, 44, 68

SCC (N=35)

ID	age	cytology	HPV	Tissue ID
Sc-0364	51	ASC-H	16	
Sc-0005	49	HSIL	16	T0001
Sc-0011	32	HSIL	16	
Sc-0127	65	HSIL	33	
Sc-0134	34	HSIL	39	
Sc-0243	60	HSIL	58	
Sc-0259	43	HSIL	52	T0092
Sc-0324	42	HSIL	18	
Sc-0355	53	HSIL	16	
Sc-0360	59	HSIL	16	
Sc-0265	59	HSIL	58	
Sc-0373	40	HSIL	59	
Sc-0426	36	HSIL	16	
Sc-0136	35	NILM	negative	
Sc-0038	42	SCC	16	T0043
Sc-0115	82	SCC	58	
Sc-0128	48	SCC	16	T0053
Sc-0186	58	SCC	58, 84	
Sc-0264	69	SCC	58	
Sc-0342	56	SCC	18	T0120
Sc-0357	85	SCC	33	
Sc-0042	30	SCC	negative	T0044
Sc-0261	67	SCC	16	
Sc-0327	38	SCC	16	T0115
Sc-0369	70	SCC	16	
Sc-0374	51	SCC	52, 58	
Sc-0384	76	SCC	52, 68	
Sc-0386	73	SCC	16	
Sc-0390	67	SCC	52	
Sc-0397	45	SCC	16	
Sc-0399	83	SCC	16	
Sc-0403	31	SCC	16	
Sc-0424	74	SCC	18	
Sc-0429	75	SCC	52	
Sc-0052	54	ND	16	

AD (N=19)

ID	age	cytology	HPV	Tissue ID
Sc-0015	52	AD	negative	
Sc-0029	45	AD	18	T0030
Sc-0044	47	AD	18	T0041
Sc-0056	68	AD	negative	
Sc-0061	42	AD	16	T0050
Sc-0095	48	AD	negative	T0061
Sc-0129	50	AD	16	T0076
Sc-0172	35	AD	16	
Sc-0354	67	AD	negative	
Sc-0022	38	AD	16	
Sc-0144	29	AD	18	
Sc-0152	67	AD	18	
Sc-0395	58	AD	16	
Sc-0413	56	AD	negative	
Sc-0436	35	AD	16	
Sc-0298	29	AGC	16, 53	
Sc-0071	60	SCC	59	T0054
Sc-0137	35	SCC	18	T0068
Sc-0251	46	ND	18	T0103

Note : CIN; cervical intraepithelial neoplasia, SCC; squamous cell carcinoma, AD; adenocarcinoma. Tissue ID corresponded to the number in figure 4A.

Supplementary Table S2. Diagnostic concordance rate with expression of miRNAs

Identification of miRNAs in cervical mucus as a novel diagnostic marker for cervical neoplasia

Satoshi Kawai, Takuma Fujii, Iwao Kukimoto, Hiroya Yamada, Naoki Yamamoto, Makoto Kuroda, Sayaka Otani, Ryoko Ichikawa, Eiji Nishio, Yutaka Torii, Aya Iwata

	126-3p	20b-5p	451a	144-3p
126-3p				
normal vs SCC	-	0.859 (0.750-0.968)	0.930 (0.852-1.008)	0.861 (0.753-0.968)
normal vs SCC+AD	-	0.833 (0.728-0.937)	0.908 (0.830-0.987)	0.854 (0.757-0.951)
20b-5p				
normal vs SCC	-	-	0.835 (0.717-0.952)	0.859 (0.750-0.968)
normal vs SCC+AD	-	-	0.815 (0.705-0.924)	0.762 (0.640-0.883)
451a				
normal vs SCC	-	-	-	0.930 (0.852-1.008)
normal vs SCC+AD	-	-	-	0.945 (0.884-1.006)

Agreement of miRNA results with cut off value between paired miRNAs was evaluated with the Cohen’s kappa(k) coefficient.

Agreement was interpreted as poor (k<0.200), weak (0.200<k<0.400), moderate(0.401<k<0.600), strong(0.601<k<0.800), near perfect(0.801<k<1.000) and perfect(k=1.000).

Supplementary Table S3. Microarray results of miR-144-5p and -4732-5p

Identification of miRNAs in cervical mucus as a novel diagnostic marker for cervical neoplasia

Satoshi Kawai, Takuma Fujii, Iwao Kukimoto, Hiroya Yamada, Naoki Yamamoto, Makoto Kuroda, Sayaka Otani, Ryoko Ichikawa, Eiji Nishio, Yutaka Torii, Aya Iwata

miRNAs	Microarray					Microarray					Realtime RT-PCR				
	global normalization (absolute value)					Fold-change(disease/normal)					Fold-change(disease/normal)				
group category	Normal	CIN1	CIN3	SCC	AD	Normal	CIN1	CIN3	SCC	AD	Normal	CIN1	CIN3	SCC	AD
hsa-miR-144-5p	not detect	2.7	6.0	59.5	200.0	not determined	not determined	not determined	not determined	not determined	not determined	not determined	not determined	not determined	not determined
hsa-miR-4732-5p	866.8	741.7	928.8	2279.3	1525.8	1.0	0.9	1.1	2.6	1.8	not determined	not determined	not determined	not determined	not determined
